
JOURNAL INFORMATION
==============================
NLM Title Abbreviation: Exp Physiol
Journal ID: Exp Physiol
NLM Journal ID: 9002940
Journal ID: EPH

Experimental Physiology

ISSN: 0958-0670
EISSN: 1469-445X
Publisher: Wiley

ARTICLE INFORMATION
==============================
PMCID: PMC12756860
DOI: 10.1113/eph.70164
Article ID: EPH70164
Article version: 1
Subjects: Issue Information, Issue Information

Issue Information

Electronic publication date: 2026 Jan 1
Publication date: 2026 Jan
Volume: 111
Issue: 1
First page: 314
Last page: 319
Copyright: © 2025 The Authors. Experimental Physiology published by John Wiley & Sons Ltd on behalf of The Physiological Society.
License: This is an open access article under the terms of the http://creativecommons.org/licenses/by/4.0/ License, which permits use, distribution and reproduction in any medium, provided the original work is properly cited.
License URL: https://creativecommons.org/licenses/by/4.0/

Figure count: 0
Table count: 0
Page count: 6

source-schema-version-number: 2.0
cover-date: 1 January 2026
details-of-publishers-convertor: Converter:WILEY_ML3GV2_TO_JATSPMC version:6.6.6 mode:remove_FC converted:01.01.2026

==============================

### Experimental Physiology

Open Access

Integration and Translation

Volume 111 | Issue 1 | 1 January 2026

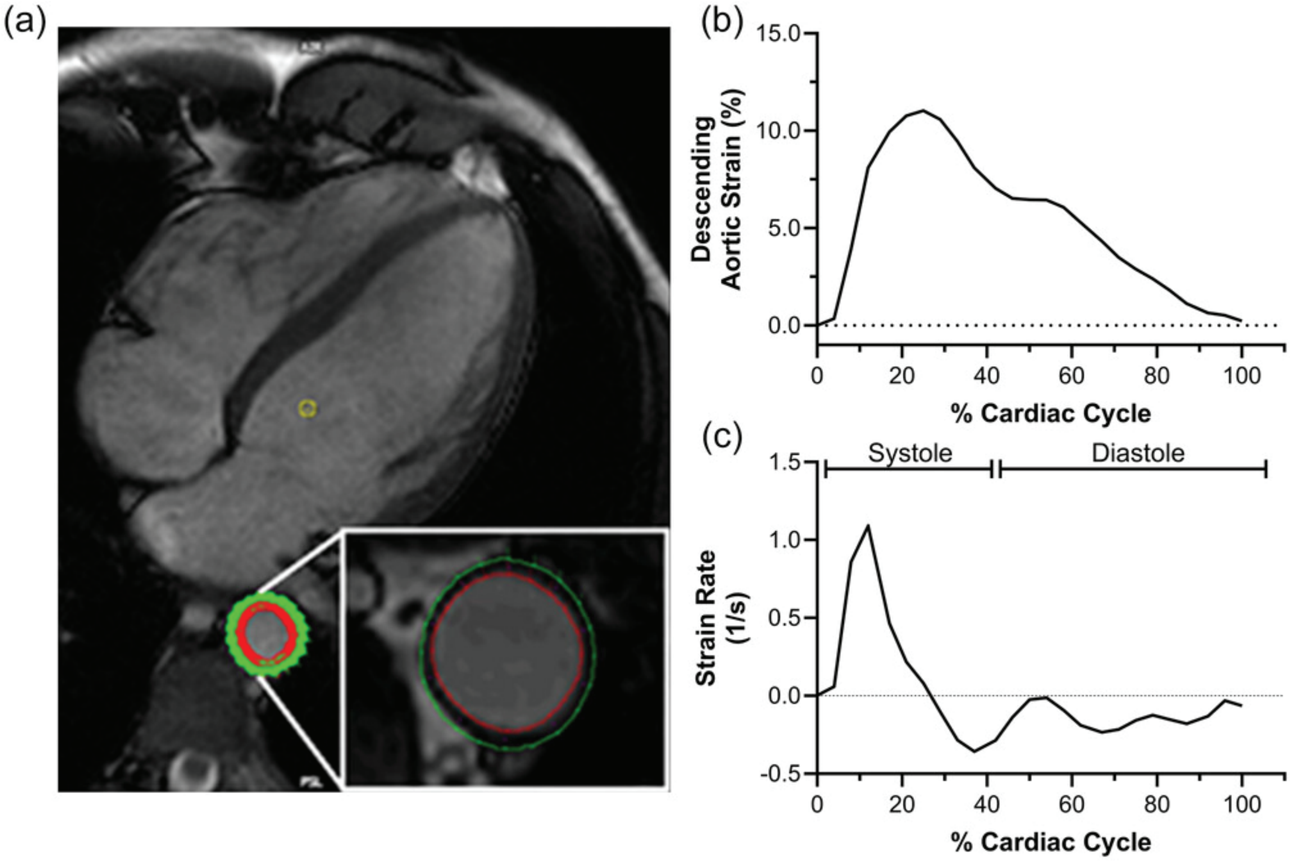

WILEY

The Physiological Society

150 YEARS
1876 2026
LEADING DISCOVERY SCIENCE

### Experimental Physiology

Open Access

Integration and Translation

Volume 111/Issue 1/
1 January 2026

### EDITORIAL BOARD

**EDITOR-IN-CHIEF**
Damian Bailey *Pontypridd, UK*

**DEPUTY EDITOR-IN-CHIEF EUROPE**
Ronan Berg *Copenhagen, Denmark*

**DEPUTY EDITOR-IN-CHIEF USA**
David Poole *Manhattan, KS, USA*

**SENIOR ETHICS EDITOR**
Susan Barman *East Lansing, MI, USA*

**STATISTICAL EDITOR**
Cody Durrer *Copenhagen, Denmark*

**SENIOR REVIEWS EDITOR**
Toby Mündel *Ontario, Canada*

**CONNECTIONS EDITOR**
Prem Kumar *Birmingham, UK*

**SENIOR EDITORS**
Dana Almohazey *Dammam, Saudi Arabia*
Jill Barnes *Madison, WI, USA*
R. Matthew Brothers *Arlington, TX, USA*
Kirstene Callø *Copenhagen, Denmark*
Ellen Dawson *Liverpool, UK*
Colleen Deane *Southampton, UK*
Katherine Denton *Melbourne, Australia*
David Edwards *Newark, DE, USA*
Peter Zu Eulenburg *Munich, Germany*
Federico Formenti *London, UK*
Julien Ochala *Copenhagen, Denmark*
Shigehiko Ogoh *Kawagoe, Japan*
Jason Peart *Gold Coast, Australia*
Peter Rasmussen *Copenhagen, Denmark*
Lee Romer *London, UK*
Harry B. Rossiter *Los Angeles, CA, USA*
Bill Sheel *Vancouver, Canada*
Mike Stembridge *Cardiff, UK*
Francisco Villafuerte *San Martín de Porres, Peru*

**REVIEWING EDITORS**
Tatsuro Amano *Niigata, Japan*
Claire Badenhorst *Palmerston North, NZ*
Tracy Baynard *Boston, MA, USA*
Kanokwan Bunsawat *Salt Lake City, UT, USA*
Hannah Caldwell *Copenhagen, Denmark*
Havovi Chichger *Cambridge, UK*
Silvia Conde *Lisbon, Portugal*
Joseph Costello *Portsmouth, UK*
Judy Delp *Tallahassee, FL, USA*
Aimee Drane *Cardiff, UK*
Esther Dupont-Versteegden *Lexington, KY, USA*
Andrea Fellet *Buenos Aires, Argentina*
Carrie Ferguson *Torrance, CA, USA*
Igor Fernandes *Niteroi, Brazil*
Ryan Hoiland *Kelowna, Canada*
Seth Holwerda *Kansas City, KS, USA*
Anna Hudson *Adelaide, Australia*
Matthew Kay *Washington, DC, USA*
James Lang *Ames, Iowa, USA*
Jacqueline Limberg *Columbia, MO, USA*
Fiona McBryde *Auckland, NZ*
Carmen De Miguel *Birmingham, AL, USA*
Niels Olesen *Copenhagen, Denmark*
Leah Reznikov *Gainesville, FL, USA*
Sarah Short *Iowa City, IA, USA*
Nathaniel Szewczyk *Nottingham, UK*
Can Ozan Tan *Boston, MA, USA*
Mike Tymko *Vancouver, Canada*
Kai Yang *Guangzhou, China*
Song Yao *Melbourne, Australia*
Gensheng Zhang *Hangzhou, China*

**FUTURE LEADERS**
Sophie Broome *Melbourne, Australia*
Callum Brownstein *Newcastle, United Kingdom*
Daniel Craighead *Minneapolis, MN, USA*
Fabio Sarto *Padua, Italy*

**CONSULTANT EDITORS**
Richard Burden *Bisham, UK*
Nic Green *Bedfordshire, UK*
Julie Greeves *Salisbury, UK*
Denis Noble *Oxford, UK*
Nanduri Prabhakar *Chicago, IL, USA*

**MANAGING EDITOR**
Alex Stewart (astewart@physoc.org)

**DEPUTY MANAGING EDITOR**
Joshua Hersant (jhersant@physoc.org)

**PEER REVIEW COORDINATOR**
Diana Jones (ephjournal@physoc.org)

**EDITORIAL OFFICE**
The Physiological Society
Hodgkin Huxley House
30 Farringdon Lane, Clerkenwell
London EC1R 3AW

**PRODUCTION OFFICE**
K. Nandini (EPH@wiley.com)

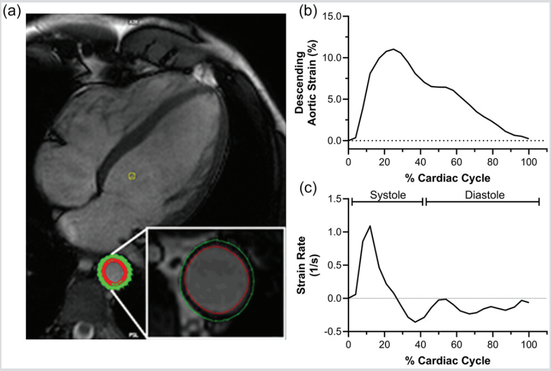

**Cover Illustration**
Circumferential strain of the descending aorta. See Wakeham et al. Pages 36–42.

**Open Access and Copyright**
All articles published by Experimental Physiology [submitted after 18 October 2022] are fully open access: immediately freely available to read, download, and share. All articles [accepted from 18 October 2022] are published under the terms of a Creative Commons license.
Copyright on any research article published by Experimental Physiology is retained by the author(s).
Further information about open access licenses and copyright can be found at: https://authorservices.wiley.com/author-resources/Journal-Authors/licensing/licensing-info-faqs.html#4. Information on the licenses used by Experimental Physiology can be found at: https://physoc.onlinelibrary.wiley.com/journal/1469445X.

For submission instructions and all other information visit: https://physoc.onlinelibrary.wiley.com/journal/1469445X

The Physiological Society and The Physiological Society logo are trade marks belonging to The Physiological Society and are registered in the UK and in the EU Community, respectively.

*Experimental Physiology* publishes high quality, original, physiological research papers that give novel insights into fundamental homeostatic and adaptive responses in health and papers that further our knowledge of pathophysiological mechanisms in diseases.

Experimental Physiology
Open Access
Integration and Translation

### INFORMATION AND GUIDANCE FOR AUTHORS

### Full version online at http://ep.msubmit.net/cgi-bin/main.plex?form_type=display_requirements

### ORIGIN

*The Quarterly Journal of Experimental Physiology*, founded in 1908 by Sir Edward Sharpey-Schafer, Professor of Physiology, University of Edinburgh, became a publication of The Physiological Society in 1981. The title was changed to *Experimental Physiology* in 1990.

### SCOPE

*Experimental Physiology* publishes research papers that report novel insights into homeostatic and adaptive responses in health, as well as those that further our understanding of pathophysiological mechanisms in disease. We encourage papers that embrace the journal's orientation of translation and integration, including studies of the adaptive responses to exercise, acute and chronic environmental stressors, growth and aging, and diseases where integrative homeostatic mechanisms play a key role in the response to and evolution of the disease process. Examples of such diseases include hypertension, heart failure, hypoxic lung disease, endocrine and neurological disorders. We are also keen to publish research that has a translational aspect or clinical application. Comparative physiology work that can be applied to aid the understanding human physiology is also encouraged.

Manuscripts that report the use of bioinformatic, genomic, molecular, proteomic and cellular techniques to provide novel insights into integrative physiological and pathophysiological mechanisms are welcomed.

We are also interested in methodological papers reporting important new developments in physiological techniques that could lead to significant new insights.

Papers which observe a significant new physiological behaviour or phenomenon which has the potential to alter the understanding of that field, but which have not yet identified the mechanisms may also be considered. Such papers should demonstrate the experimental examination and exclusion of a number of plausible mechanisms for the important novel phenomenon.

**Article Types**

In addition to original research, *Experimental Physiology* consider the following article types for publication:

*Case studies, Short Communication, Connections, Reviews, Full Reviews, Hot Topic Reviews Myths and Methodologies and Symposium Reports.*

Review articles are normally by invitation but proposals may be submitted for consideration.

Commentary articles in the form of Viewpoints and Letters are also published.

For full guidelines on all published article see full guidelines.

C O P E COMMITTEE ON PUBLICATION ETHICS

This journal is a member of and subscribes to the principles of the Committee on Publication Ethics

### ETHICS OF EXPERIMENTATION

**Animal Experiments** - see full journal policy at https://physoc.onlinelibrary.wiley.com/hub/animal-experiments

Experimental Physiology endorses the ARRIVE Guidelines for reporting *in vivo* experiments: http://onlinelibrary.wiley.com/doi/10.1113/expphysiol.2010.053793/full

Authors of papers submitted to Experimental Physiology must adhere to the journals' principles and standards for reporting animal experiments.

Authors should read these guidelines to ensure that their research and experiments adhere to our exacting standards.

Euthanasia/humane killing: Animals must be killed using methods approved for that species, stage of development and size. The standards required by the journals are those of the UK/European Union legislation, set out in in ASPA Schedule 1 in the UK and in Annex IV in the European Directive 2010/63/EU. Methods not on Schedule 1 or Annex IV could be permissible but even where the method used conforms with national guidelines, the onus is on the author to provide scientific evidence to demonstrate that it is at least as humane as those on Schedule 1 and Annex IV.

Our guidelines have been outlined in the Editorial 'Principles and standards for reporting animal experiments in Experimental Physiology'.

**Confirmation of compliance**

Authors must state in the Methods section that the investigators understand the ethical principles under which the journal operates and that their work complies with the animal ethics checklist as outlined in the Editorial.

**Human Experiments** - See full journal policy at https://physoc.onlinelibrary.wiley.com/hub/human-experiments

The Editorial Boards are concerned that all work published in The Journal of Physiology and Experimental Physiology should have been conducted according to the highest standards of safety and ethics. Legislation and accepted practice concerning human experimentation vary from country to country and consequently it is difficult to define absolute requirements. However, work with human subjects should conform to the standards set by the Declaration of Helsinki (last modified in 2013), the Medical Research Council's online guidance on the use of human tissue, and the guidelines set out below. The objectives must be to ensure that all risks are minimized and that subjects are not injured and do not feel they have been abused as a result of participating in the study. Any definition of abuse will include excessive or unexpected pain or discomfort experienced during the experiment. Note, in the case of experiments involving minors, any risks must have been considered absent or minimal, and evidence must be presented that the experiments were performed with the understanding and consent of the legal guardian.

All manuscripts must contain statements indicating that (i) informed consent has been obtained, preferably in writing, (ii) studies conformed to the standards set by the Declaration of Helsinki or the version that was in place at the time of the experiments, NOTE: If the research study was registered (clause 35 of the Declaration of Helsinki) the registration database should be indicated, else the lack of registration should be noted as an exception (e.g. The study conformed to the standards set by the Declaration of Helsinki, except for registration in a database.); and (iii) the procedures have been approved by the local ethics committee.

### DISCLAIMER

The Publisher, the Physiological Society and Editors cannot be held responsible for any errors in or any consequences arising from the use of information contained in this journal. The views and opinions expressed do not necessarily reflect those of the Publisher, the Physiological Society or Editors, neither does the publication of advertisements constitute any endorsement by the Publisher, the Physiological Society, Editors, or Authors of the products advertised.

Wiley Open Access articles posted to repositories or websites are without warranty from Wiley of any kind, either express or implied, including, but not limited to, warranties of merchantability, fitness for a particular purpose, or non-infringement. To the fullest extent permitted by law Wiley disclaims all liability for any loss or damage arising out of, or in connection with, the use of or inability to use the content.

### Experimental Physiology

Vol 111.1 **Contents of volume 111.1** 1 January 2026

### REVIEW ARTICLE

### 2024 MID-CAREER RESEARCHER PRIZE WINNER

1 The multifaceted roles of ketones in physiology
*Seyed Amirhossein Tabatabaei Dakhili, Kunyan Yang, Magnus J. Stenlund and John R. Ussher*

### EDITORIAL

14 Status quo of *Experimental Physiology* – Anno 2025: What's in a name?
*Damian M. Bailey, David C. Poole and Ronan M. G. Berg*

21 From blood flow to organ function: The physiology of autoregulatory dynamics
*Niels-Henrik Holstein-Rathlou and Donald J. Marsh*

26 One hundred years of change?
*Martha C. Tissot van Patot*

### VIEWPOINT

28 Size, more than oxygen need, determines the number of capillaries around a muscle fibre
*Christopher S. Fry and Carlo Reggiani*

30 The search for the ultra-elusive: Can computed cardiopulmonography enhance early detection of gas exchange abnormality?
*Harry B. Rossiter and Yannick Molgat-Seon*

33 A slow burn: Revisiting cross-adaptation through long-term heat acclimation
*Elliott J. Jenkins and Michael Stembridge*

### SHORT COMMUNICATION

36 Circumferential strain and strain rates of the descending aorta as novel measures of aortic stiffness and wall mechanics from standard cardiac MRI
*Denis J. Wakeham, Sauyeh K. Zamani, Andrew P. Oneglia, Matthew M. Howrey, Samer Majeed, Tiffany L. Brazile, Joshua A. Beckman, James P. MacNamara, Mark J. Haykowsky, Vlad G. Zaha, Benjamin D. Levine, Christopher M. Hearon Jr, Satyam Sarma and Michael D. Nelson*

### REVIEW ARTICLE

43 Aortic and cardiovascular remodelling after thoracic endovascular aortic repair for blunt traumatic aortic injury in younger patients: A narrative review of physiological and clinical outcomes
*Marco David Bokobza De la Rosa, Matti Jubouri, Thurkga Moothathamby, Mohamed Refaie, Ali Murtada, Idhrees Mohammed, Ian M. Williams, Damian M. Bailey and Mohamad Bashir*

### RESEARCH ARTICLE

### CARDIOVASCULAR CONTROL

54 Inhibitory effect of oestradiol on the cardiac $K_V$7.1/KCNE1 channel is species dependent
*Veronika A. Linhart, Lucas Dauga and Sara I. Liin*

### ENCRODINOLOGY AND METABOLISM

69 Effects of short-term exercise on plasma metabolic and lipidomic profiles of individuals with type 2 diabetes
*Maartje Cox, Aaron Raman, Timothy Fairchild, John P. Beilby, Bu B. Yeap, Jeremy K. Nicholson, Julien Wist, Jeremiah Peiffer and Nathan G. Lawler*

98 Acute alterations in blood lactate in the setting of transient stress induced myocardial ischaemia
*Jamie M. O'Driscoll, Elliot Smith, Matchel Bibat, Jamie J. Edwards, Claire Compton, Konstantina Kipourou, Damian Coleman, Jonathan Wiles, Eliane Cunliffe, Anna Marciniak and Rajan Sharma*

111 Topical application of the cold-mimetic L-menthol decreases wheel running without affecting the beneficial effects of voluntary exercise in mice
*Annalaura Bellucci, Bradley J. Baranowski, Stewart Jeromson, Michael Akcan, Serena Trang, Meagan Arbeau, Hadil Alfares, Katelyn Eisner and David C. Wright*

### ENVIRONMENTAL AND EXERCISE

124 Heat to hypoxia cross-adaptation: Effects of 6-week post-exercise hot-water immersion on exercise performance in acute hypoxia
*Patrick Rodrigues, Lydia L. Simpson, Justin S. Lawley, Heru S. Lesmana and Anne Hecksteden*

139 Improving coordination, proprioception, balance and motor proficiency in Down syndrome with developmental games
*Alireza Rezaee, Hasan Daneshmandi, Hesam Ramezanzade, Sahar Mohammadzadeh, Mert Kurnaz and Mustafa Altınkök*

153 Physical exercise intervention in glycogen storage disease IIIa: Feasibility and multisystem benefits
*Asunción Bustos-Sellers, Almudena Montalvo-Pérez, Araceli Boraita, Gonzalo Saco-Ledo, Lidia B. Alejo, David Barranco-Gil, Pedro L. Valenzuela, Nerea López-Maldonado, Roser Ferrer-Costa, Javier Esparcia-Estrela, Celia Monteagudo-López, Tomàs Pinós, Alejando Santos-Lozano, Alejandro Lucia and Alfredo Santalla*

167 Increases in cardiac vagal modulation following muscle mechanoreflex activation via passive calf stretch: Impact of interindividual differences
*Georgia C. S. Lehnen, Marcela S. Araujo, Igor S. Rocha, Jeann L. Sabino-Carvalho, Rosa V. D. Guerrero, Gabriel S. Trajano and Lauro C. Vianna*

### HEART/CARDIAC MUSCLE

179 Bioenergetic profiles and respiratory control in mitochondrial physiology: Precision analysis of oxidative phosphorylation
*Alba Timón-Gómez, Carolina Doerrier, Zuzana Sumbalová, Luiz F. Garcia-Souza, Eleonora Baglivo, Luiza H. D. Cardoso and Erich Gnaiger*

### MUSCLE

212 Diffusion and physical constraints limit oxidative capacity, capillary supply and size of muscle fibres in mice and humans
*Hans Degens, Guy A. M. Messa, Jason Tallis, Alessandra Bosutti, Tomas Venckunas, Ismail Adeniran, Rob C. I. Wüst and Paul W. Hendrickse*

226 Exercise modality-dependent mitochondrial respiratory capacity in satellite cells and conditioned serum-induced responses in cultured myotubes
*Takanaga Shirai, Hayato Shinkai, Riku Tanimura, Kazuki Uemichi, Shunsuke Sugiyama, Kohei Takeda, Yu Kitaoka and Tohru Takemasa*

### NEUROSCIENCE

240 Visual and vestibular reweighting after cyber- and space-sickness
*Tess Bonnard, Emilie Doat, Jean-René Cazalets, Dominique Guehl and Etienne Guillaud*

257 The role of orexin A in the pathogenesis of ischaemic stroke at high altitude
*Minxia Zhu, Xu Guo, Jing Guo, Zhaojun Zhang, Kexin Zhang, Yaqi Lei, Yuhe Wang and Haoshuang Zhan*

### RESPIRATORY

270 Physiology of nitrogen: A life or death matter
*Damian M. Bailey, Vaughan G. Macefield and David C. Poole*

283 Ageing exacerbates the adverse effects of respiratory muscle fatigue on vascular function, locomotor muscle fatigue and exercise performance in males
*Valentin Mons, Colin Lavigne, Olivier Meste, Benjamin Mauroy and Gregory M. Blain*

300 Computed cardiopulmonography: Effects of physical characteristics on lung parameter estimates
*Asma Alamoudi, Dominic Sandhu, Thomas D. E. Bithell, Nicholas M. J. Smith, Graham Richmond, Lorenzo S. Petralia, Snapper Magor-Elliott, Haopeng Xu, Nick P. Talbot, Grant A. D. Ritchie, Nayia Petousi and Peter A. Robbins*

Editor's Pick

Circumferential strain of the descending aorta. See Wakeham et al. Pages 36–42.

Reasons to submit to

### Experimental Physiology

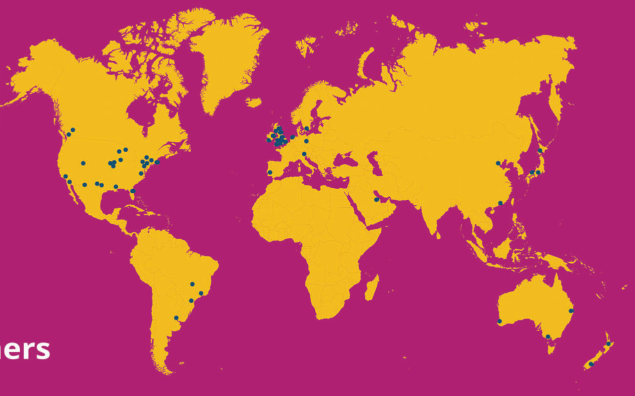

**Annual prizes for early and mid-career researchers**

**68 Board Members from 14 counties**

Published research from **24** countries in 2024

**Publishes articles in all areas in physiology.** Publishes novel insights into homeostatic and adaptive responses in health and disease.

OPEN

### Get your work seen, read and cited with Open Access publication.

8 years

Cited half-life of over 8 years

Over 1 million downloads per year

32

An average of 32 days from submission to first decision with two reports

Acceptance to online publication within 5 days

No page or figure limits

Pre-submission enquiries encouraged ephjournal@physoc.org

Prior publication on preprint servers allowed

1/3

46% of articles accepted in 2024

14 Nobel Prize winners

**2.6**
2023 Impact Factor

Editor-in-Chief
**Damian Bailey**
University of South Wales, UK

ephjournal@physoc.org

**ep.physoc.org**

**bit.ly/ExpPhysiolLinkedIn**

**/expphysiol**

**@ExpPhysiol**

WILEY
